# Apparent climate-mediated loss and fragmentation of core habitat of the American pika in the Northern Sierra Nevada, California, USA

**DOI:** 10.1371/journal.pone.0181834

**Published:** 2017-08-30

**Authors:** Joseph A. E. Stewart, David H. Wright, Katherine A. Heckman

**Affiliations:** 1 Department of Ecology and Evolutionary Biology, University of California, Santa Cruz, CA, United States of America; 2 California Department of Fish and Wildlife, North Central Region, Rancho Cordova, CA, United States of America; 3 USDA Forest Service, Northern Research Station, Lawrence Livermore National Lab, Livermore, CA, United States of America; University of Colorado, UNITED STATES

## Abstract

Contemporary climate change has been widely documented as the apparent cause of range contraction at the edge of many species distributions but documentation of climate change as a cause of extirpation and fragmentation of the interior of a species’ core habitat has been lacking. Here, we report the extirpation of the American pika (*Ochotona princeps*), a temperature-sensitive small mammal, from a 165-km^2^ area located within its core habitat in California’s Sierra Nevada mountains. While sites surrounding the area still maintain pikas, radiocarbon analyses of pika fecal pellets recovered within this area indicate that former patch occupancy ranges from before 1955, the beginning of the atmospheric spike in radiocarbon associated with above ground atomic bomb testing, to c. 1991. Despite an abundance of suitable rocky habitat climate warming appears to have precipitated their demise. Weather station data reveal a 1.9°C rise in local temperature and a significant decline in snowpack over the period of record, 1910–2015, pushing pika habitat into increasingly tenuous climate conditions during the period of extirpation. This is among the first accounts of an apparently climate-mediated, modern extirpation of a species from an interior portion of its geographic distribution, resulting in habitat fragmentation, and is the largest area yet reported for a modern-era pika extirpation. Our finding provides empirical support to model projections, indicating that even core areas of species habitat are vulnerable to climate change within a timeframe of decades.

## Introduction

A dominant conceptual framework for understanding the impacts of climate change on species focuses on the widely documented pattern of poleward and upslope range shifts, exemplified by peripheral range contraction and expansion [1–3]. Some studies provide counter-examples wherein a minority of species—perhaps sensitive to different seasonal or hydrological aspects of climate—have experienced distributional shifts counter to the predominant direction [4–6]. Theoretical work has highlighted how metapopulation structure, local adaptation, and variable habitat quality may interact with climate change to cause fragmentation and extirpation of internal, non-peripheral habitat [7–10]. Similarly, empirical work has documented internal extirpations and fragmentation associated with species declines, but not related to climate change [11,12]. Documentation of climate-mediated fragmentation of habitat internal to an area of a species’ contiguous distribution has been lacking (but see [13]).

The American pika (*Ochotona princeps*, hereafter “pika”) is a temperature-sensitive lagomorph species and habitat specialist, which lives primarily in talus habitat (broken rock debris fields) throughout western North America. The species is adapted to cold climates, and possesses a thick coat of fur and limited tolerance for elevated body temperatures, which it regulates behaviorally [14–16]. Paleontological records of distribution show pikas shifting up or down in elevation during periods of climate warming or cooling, respectively [17–20]. The influence of climate-driven range fluctuation has also been observed in pika population genetics [21]. Authors working in the Great Basin, southern Utah, and California, USA, have identified distributional shifts consistent with a response to changes in temperature induced by anthropogenic global warming [22–27], though examples of pikas living in relatively warm, low-elevation sites have also been presented [28–31].

In contrast to the sky islands of the Great Basin, pikas have until recently seemed relatively secure in their core areas: areas that might be considered “mainlands” of pika habitat and distribution, such as the Sierra Nevada range of California. Climate change could be seen as eroding the edges of the pika’s Sierra Nevada distribution, but persistence within higher, cooler elevations has appeared strong [26,27,32,33].

We present new information that provides a less sanguine assessment of the pika’s security within core areas of its range in the Sierra Nevada. While previous studies have documented the extirpation of pikas from 3-km-radius (28 km^2^) or smaller areas in the Great Basin or Sierra Nevada [22–27], here we report the extirpation of the pika from an unprecedentedly large (165 km^2^) region within a broad contiguous area of its distribution in the Sierra Nevada. Pika fecal pellets, readily recovered from nearly every talus patch searched within the area, provide evidence of widespread former occupancy. Given documented temperature changes in our study area and the physiology, behavior, and prehistoric and historical shifts in distribution of pikas in response to climate, we conclude that climate warming is well-supported as a driver of this extirpation.

## Methods

### Study area

**Fig 1:**
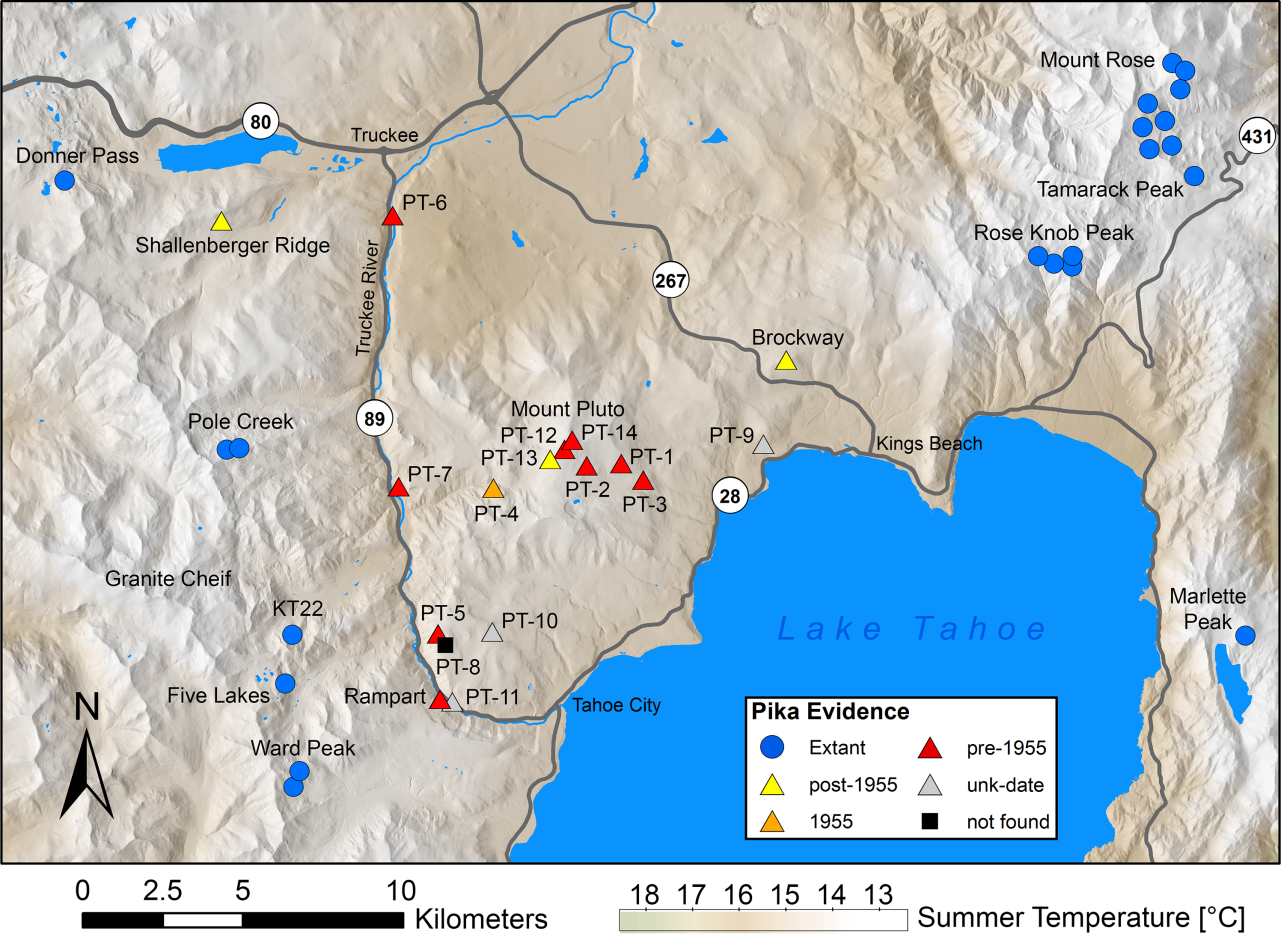
Sites surveyed for American pikas in the north Lake Tahoe area, California, USA. The Pluto triangle is bounded by Lake Tahoe, highway 267, and by the Truckee River, adjacent to highway 89. Current sign of American pika was found both east and west of Pluto triangle; nearly all sites within the Pluto triangle yielded old fecal pellets (see also S1 Table). Map boundaries represent our boundaries for the north Lake Tahoe area.

Pika surveys were conducted throughout the north Lake Tahoe area with a particular focus on the roughly triangular area surrounding Mt. Pluto, which we refer to as the Pluto triangle (Fig 1, S1 Table). The Pluto triangle is bounded by Lake Tahoe, the Truckee River, and Highway 267, with vertices approximately at the towns of Truckee, Kings Beach, and Tahoe City, California. Pika dispersal to the region from surrounding pika-habitable areas may be constrained by Lake Tahoe to the southeast, the Truckee River (elevation 1900 to 1760 m) to the west, and lower-elevation habitats to the northeast, based on known factors affecting pika dispersal [34,35]. According to Hafner’s equation [17] adjusting pika-suitable elevations by latitude and longitude, 77% of the Pluto triangle’s extent is within pika-suitable elevations (above 1896 to 1916 m). Habitable talus areas are naturally more restricted than this, but occur in considerable expanses on and around Mount Pluto (2625 m) and Mount Watson (2568 m) and along the east slope of the Truckee River canyon. Major areas of suitable habitat adjacent to the triangle include the Carson Range less than 10 km east and the Sierra Nevada crest as little as 4 km west of the Truckee River. Brockway Summit (2194 m) is the highest pass connecting the Pluto triangle to other habitable areas, in this case, the Carson Range. Highways 89 and 267 are two-lane, paved, high-speed roads with moderate daytime traffic.

Within the Pluto triangle, during 2011–2016 we surveyed talus habitats of good apparent suitability for American pika, based on our experience of pika habitat quality and published assessments of habitat factors (e.g., rock size) [36]. Additional surveys also were carried out by authors and colleagues at talus patches outside the triangle (Fig 1, S1 Table). Talus patches were located using aerial imagery and in situ observation. We hand-delineated all talus within the Pluto triangle and all talus within 1 km of survey sites outside the Pluto triangle into a GIS layer using the best available aerial imagery (Google Earth, typical resolution in our study area: 0.15 m). Pika surveys within the Pluto triangle sampled within all areas of pika habitat in the triangle, with emphasis on the most suitable regions (higher elevations, larger talus patches) [24,27]. Within each area of pika habitat we selected sites for survey with the highest apparent habitat suitability based on our experience of pika occurrence in the northern Sierra Nevada. Some areas delineated as talus with aerial imagery proved to be marginal in terms of suitable rock sizes (i.e., < 20 cm in maximum dimension) when approached on the ground, and were not surveyed. Overall effort to detect pikas within suitable habitat in the Pluto triangle consisted of 127 person-hours of search effort.

### Surveys

We surveyed for signs of past and present pika occupancy at all study sites using methods modified from previously published research [26,27]. Briefly, transects across talus patches were walked in a zig-zag pattern by one or more observers, looking for fecal pellets, haypiles, or pikas, and listening for pika calls. Fecal pellets were considered recent if they contained green hues from undecomposed chlorophyll. A bright flashlight or mirror was used to brighten dark crevices among the rocks in order to locate pika evidences. Each site was surveyed, on one or more occasion, for a minimum of one person-hour or until all talus habitat was searched. Pikas are highly detectable, based largely upon their frequent calls and copious and persistent fecal pellets. Previous studies indicate consistently high detection probability (> 0.9) after 15–30 min of search effort [23,29,32,37]. As additional measures to confirm extirpation, at sites of high apparent suitability and at the highest elevation sites within the Pluto triangle, we conducted listening surveys during crepuscular hours, when pikas are most vocal, and camped adjacent to talus fields to listen for their calls.

This paper uses surveys for buried relict fecal pellets of American pikas. As has previously been reported [38,39], pika fecal pellets may persist for years or decades, perhaps especially when protected from moisture by dry climate and by shelter under rocks. We have discovered in numerous locations that pika fecal pellets persist below the talus surface in accumulations of duff, even when no other pika sign can be found above ground. We refer to these subsurface fecal pellets as buried. In this study, we conducted a timed search at all sites with no current evidence of pika occupancy, sifting scoops of debris and duff for buried pika fecal pellets. We searched each site for buried pellets for at least 30 person-minutes or until they were found. Site characteristics and search effort were recorded and fecal pellet samples were collected. All fecal pellet samples were collected in the service of the California Department of Fish and Wildlife, the public trust agency for state wildlife, and necessary permissions for surveys were obtained from landowners, including the US Forest Service.

### Radiocarbon-dating

Pika fecal pellets were collected in all surveys where adequate samples (≥ 10 pellets) were encountered, and were sent to the Carbon, Water & Soils Research Lab in Houghton, Michigan for pretreatment and graphitization. Samples were pretreated according to methods described in Millar et al. [39] to isolate plant fragments contained in the pellets. Samples were graphitized according to Vogel et al. [40], and measured for radiocarbon abundance at the Center for Accelerator Mass Spectrometry, Lawrence Livermore National Lab (LNLL) and the W.M. Keck Carbon Cycle Accelerator Mass Spectrometer Facility, University of California, Irvine (UCI) [41,42].

Radiocarbon data is reported as Fraction Modern (Fm) and calibrated calendar ages. Fm as a unit of measurement expresses the abundance of radiocarbon in a sample, with larger Fm values reflecting a more recent calendar age and smaller values reflecting an older calendar age. Radiocarbon activities were calculated using a standard δ^13^C ratio of -25‰ with respect to Pee Dee Belemnite [43]. For samples analysed at UCI, an inline δ^13^C correction was applied. At wildland sites similar to ours, Millar et al. [39] reported minimal effect on calculated radiocarbon dates due to variation in the δ^13^C ratio of pika fecal pellets, or due to dilution of atmospheric ^14^C due to air pollution from combustion of fossil fuels. Fm values reported here include a background subtraction determined from ^14^C-free coal and the aforementioned δ^13^C correction to account for isotopic fractionation [43]. Calibrated dates and/or date ranges for each sample were determined using the IntCal NH_zone2 calibration curve [44] appended to the IntCal 13 calibration curve [45] and the software program OxCal 4.2 [46]. Reported date ranges are 95% confidence intervals, and take into account the analytical error associated with each measurement. Pellets from a single sampling location were composited, and results thus represent an average ^14^C value and corresponding age, since we could not identify pellets deposited at a single moment.

### Historical climate change

We used data from the Tahoe City weather station, located at the southwestern vertex of the Pluto triangle, which has the most complete record of historical temperatures and snowpack in the northern Lake Tahoe region (1910–2015; accessed from http://climate.usurf.usu.edu). Detailed station history documentation (accessed from http://www.ncdc.noaa.gov/) indicated the station had high location and instrumentation fidelity. We restricted analyses to include only years with minimal missing daily data (< 15 days of missing data for annual temperature, N = 99, years excluded = [1964, 1977, 1978, 1989, 2009, 2010, 2011]; < 10 days for summer temperature [June, July, August], N = 105, year excluded = 1978) and linearly imputed missing daily values between days with recorded temperatures. Removing these same years from linear regressions of Sierra Nevada region-wide temperature over time (1910–2015, available from http://wrcc.dri.edu/monitor/cal-mon/ [47]) did not significantly alter the slopes of these regressions (Welch’s t-test, p = 0.69 for annual temperature, p = 0.98 for summer temperature). Data from other nearby weather stations were excluded due to incompleteness of their records (i.e. > 40% missing data). We evaluated historical change in temperature and snow depth with linear regression and pairwise tests. All p-values reported in this paper are for two-tailed tests.

### Modeled habitat suitability

We examined current (2001–2010) and historical (1910–1955) estimates of habitat suitability at survey locations in the north Lake Tahoe area (Fig 1, S1 Table, N = 38) using our previously published model [27] of pika site suitability, parameterized with historical revisit data for pika sites across California. The model estimates pika site suitability as a function of talus area (positive effect) and the lowest mean summer temperature (MST; negative effect) found within a 1-km radius of site centroids. We measure habitat abundance using talus area instead of talus perimeter because there is less uncertainty in this measurement (i.e. associated with how talus boundaries are delineated) and because talus area outperformed talus perimeter as a predictor of pika occupancy in our previous study [27]. Observed occupancy was compared against predicted occupancy using an exact binomial test and a 0.5 probability threshold for predicted occupancy. To test the importance of thermal refugia within long-distance dispersal thresholds for pikas, we also compared logistic models of minimum (refugial) MST within 1, 2, 3, 4, and 5 km of north Lake Tahoe area survey locations (Fig 1, S1 Table) as predictors of occupancy status [48]. Plausible dispersal thresholds were estimated from the literature [34,49,50]. The 5 refugial MST variables were evaluated in simple logistic regression and in combination with talus area for a total of 10 candidate models. Current and future temperatures were extracted from monthly, 270-m resolution, interpolated surfaces [51,52]. To reduce spatial bias, occurrence locations within 250-m of each other were reduced to one unique record location by random selection. Historical temperatures for the period 1910–1955 were estimated using the delta method [53] and weather station data from Tahoe City (base period: 2001–2010). Talus area within a 1-km radius of site centroids was log-transformed to reduce skew. We used the resulting best performing model to estimate the area of climatically suitable potential habitat for pikas within the surrounding greater Lake Tahoe area (latitude extent [38.543, 39.706], longitude extent [-120.786, -119.557]) for current (2001–2010) and future (2030 [2020–2040], 2050 [2040–2060]) periods.

## Results

### Surveys

We surveyed 14 sites within the Pluto triangle between 2011 and 2016, some on multiple occasions, and surveyed or compiled survey information for an additional 24 sites in the north Lake Tahoe area, but outside the Pluto triangle (Fig 1, S1 Table). No current occupancy of pika was found at any site within the Pluto triangle. Old pika fecal pellets were recovered, with relative ease (mean time to first detection 15 person-minutes, range 4–54), at all Pluto triangle sites except PT-8 (S1 Table). Old surface pellets were found at five Pluto triangle sites (PT-1, 3, 4, 11 and 12). No haypiles were found at Pluto triangle sites, consistent with an older origin, precluding us from using the novel technique of Millar et al. [39] to achieve higher-resolution dates.

Outside the triangle, we and others readily found evidence of current-year occupancy by pikas at taluses both east and west of the Pluto triangle: in the Carson range and along the Sierra crest (Fig 1, S1 Table). Pikas were found to be extant (by sighting, calls, green haypile, or fresh pellets) at 21 sites outside the triangle (S1 Table). Old surface or buried pellets were the only sign at three lower-elevation sites we surveyed outside the triangle.

### Radiocarbon-dating

**Fig 2:**
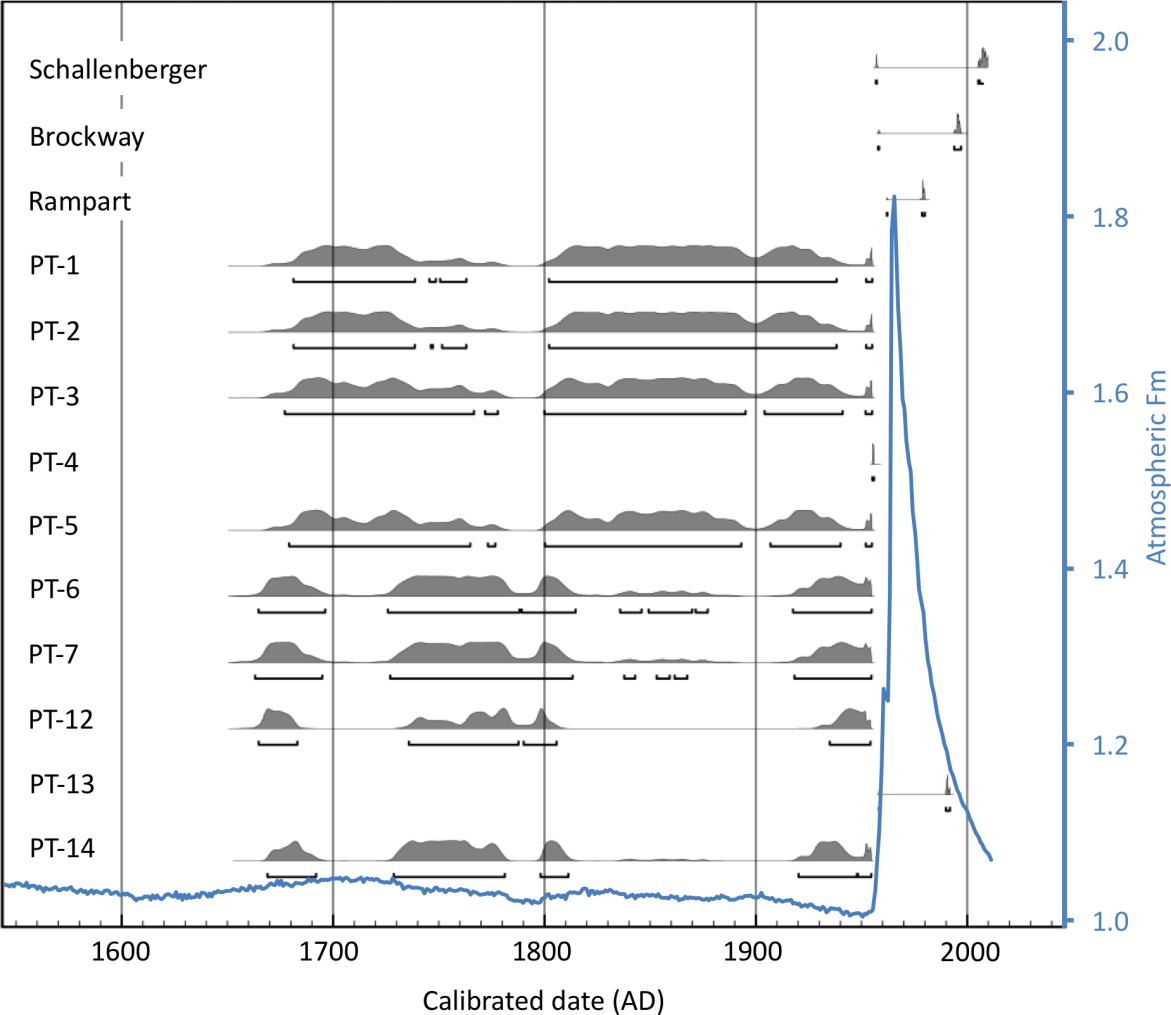
Calibrated age ranges for each of the remnant pika scat samples. The gray curves show the probability distribution of dates corresponding to each sample’s Fm estimate. Samples are stacked for comparison. The blue curve shows the radiocarbon signature of the atmosphere over time. This is the calibration curve used to estimate the ages of the relict pika scat. See text for definition of Fm units.

Calibrated ages for pika fecal pellets recovered from Pluto triangle sites ranged from pre-atomic bomb testing (prior to 1955) to 1991. Two sites within the triangle dated to the post-1955 era. Pellets from the PT-4 site had a unique radiocarbon value associated with only one calendar year on the calibration curve, 1955. Pellets from the PT-13 site had radiocarbon values that yielded an age range from 1958 to 1991. Ages for pellets from 8 Pluto triangle sites and one adjacent site dated before 1955, in a region of poor resolution on the radiocarbon calibration curve with estimated ages spanning three centuries (late 1600s to 1955) (Fig 2, S2 Table). Subtle fluctuations in radiocarbon production in the atmosphere over time lead to variations or “wiggles” in the radiocarbon calibration curve, so that a single radiocarbon value can yield multiple calendar ages. The probability distributions of calendar age ranges are represented by the gray calibration profiles in Fig 2, with 95% confidence intervals depicted in brackets below. For these 9 sites, radiocarbon dating cannot resolve an exact time of extirpation, it can only indicate that the pellets were from before c. 1955. Two of the sites examined outside the Pluto triangle, Schallenberger Ridge and Brockway Summit, yielded scat dates post-1955, suggesting that these sites were occupied more recently. We document that pika feces–with their high lignin content–can persist for more than 60 years, without preservation in caves or *Neotoma* middens [17,19,39].

### Historical climate change

**Fig 3:**
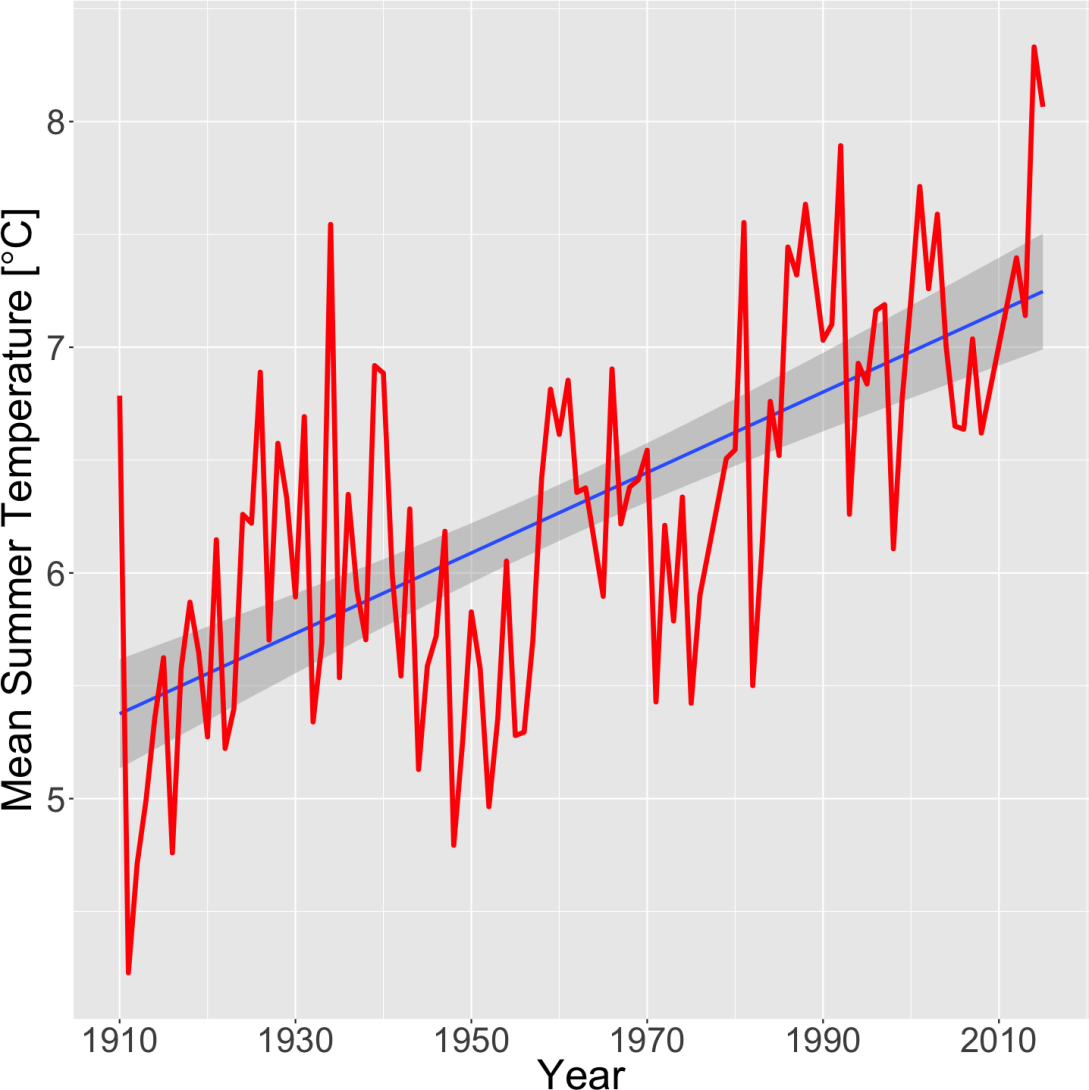
Mean annual temperature data from weather station at Tahoe City, California, USA. Temperatures have increased by 1.9°C during the period of record, 1910–2015 (linear regression, p < 10^−12^). Tahoe City forms the southwestern vertex of the Pluto triangle (Fig 1).

Mean annual temperatures (MAT) at Tahoe City have increased markedly, by 1.9°C, during the period of instrumental record, 1910–2015 (Fig 3, linear regression, p < 10^−12^, N = 99). Mean summer temperatures (MST) at Tahoe City have also increased substantially (+1.6°C, p < 10^−5^, N = 105). MAT and MST for the instrumental period following 1955 are significantly higher than for the period including and preceding 1955 (Welch’s t-test, p < 10^−8^ and p < 0.0005). Weather station data also show a marked decrease in accumulated April snow depth over the period of record for snowpack, 1911–2015 (S1 Fig; Spearman’s test, p < 0.0005, N = 91), with significantly lower snow depth in the years following 1955 than the preceding years (Mann-Whitney, p < 0.0005). The frequency of years with negligible (< 2 cm) snowpack increased from 0% of years before 1955 to 34% of years after (z-test, p < 0.0005).

### Modeled habitat suitability

Assessing the effects of the increases in MST over the period of record (1910–2015) using our previously published model of pika occupancy at historical sites in California [27] resulted in declining estimates of pika site suitability over time. Whereas 93% (13/14) of sites within the Pluto triangle were modeled to have suitable conditions (≥ 0.5 probability of occupancy) for pikas prior to 1956, that number declined to 50% (7/14) of sites under current (2001–2010) conditions (S2 Fig). Given the 100% rate of observed extirpation within the triangle, the observed extirpations substantially exceeded our previous-model [27] prediction of extirpation at these sites (exact binomial test, p < 0.001). The previous model correctly predicted current occupancy at 86% (18/21) of currently occupied sites in the north Lake Tahoe region (Fig 1), but correctly predicted extirpation at only 50% (8/16) of currently extirpated sites (< 0.5 probability of occupancy). The relatively large amount of talus area near some sites within the Pluto triangle yielded relatively high model estimates of site suitability despite relatively warm temperatures. However, the amount of talus area within 1 km of sites in the Pluto triangle was not statistically distinguishable from historical sites in California (Mann-Whitney test, p = 0.24) and 84% of historical sites in California had less surrounding talus area than the maximum talus area for sites within the Pluto triangle.

**Fig 4:**
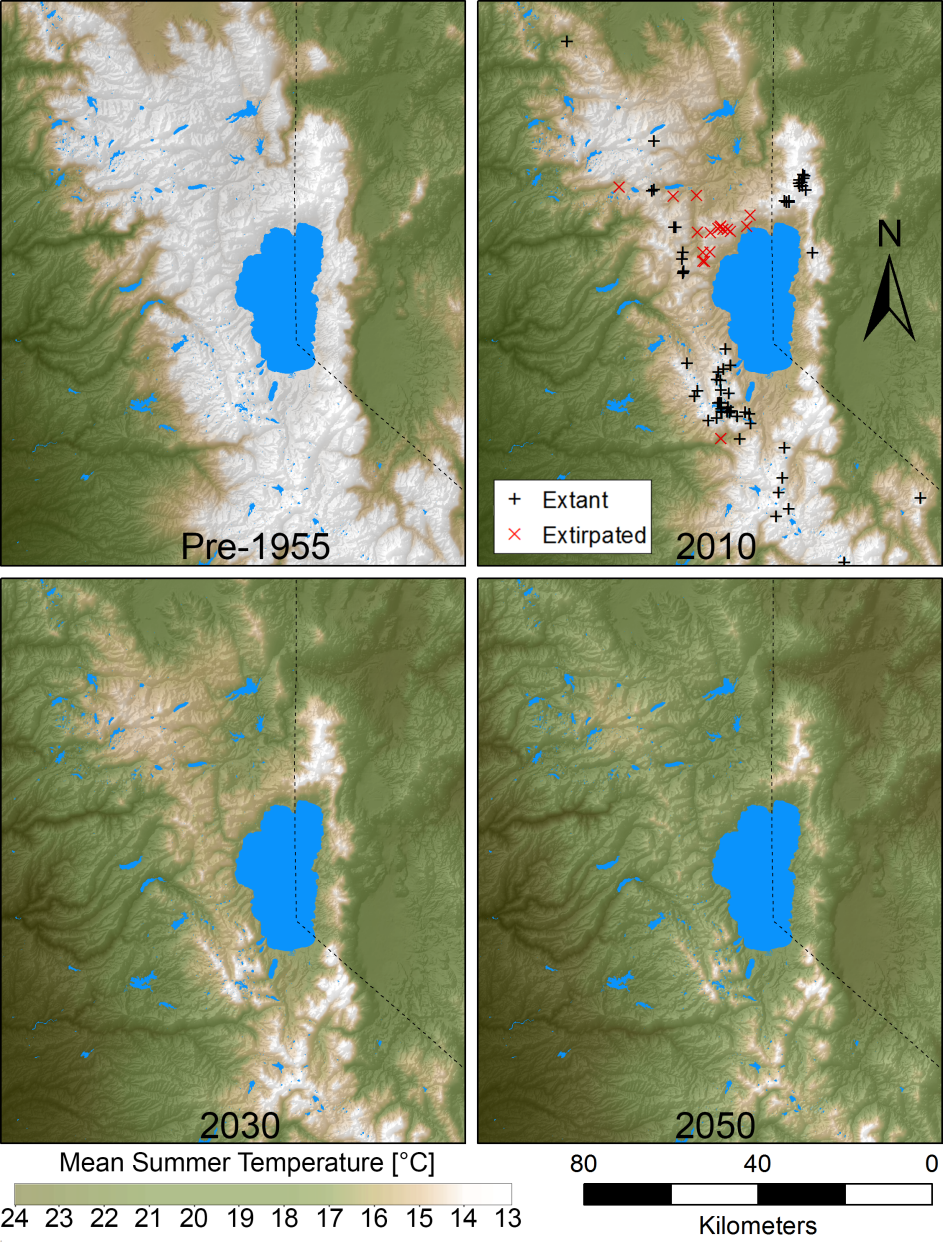
Inundation of sky islands surrounding Lake Tahoe by rising tides of warm air. Panels depict mean summer temperature (MST) for historical conditions, 1910–1955 (current MST -1.45°C), current conditions, 2010 (2001–2010), and future conditions (2030, RCP 8.5, current MST + 1.33°C; 2050, RCP 8.5, current MST + 2.74°C). Fourteen degrees Celsius MST represents an approximate threshold above which pika occupancy becomes tenuous [27]. Projections are the ensemble mean of 17 general circulation models [54]. Temperatures from the mid-20^th^ century and before (upper left panel) appear to have supported spatially continuous pika-habitable temperatures throughout much of the Tahoe region. Current temperatures (upper right) have seen the collapse of the Pluto triangle metapopulation, which occupied the isthmus of habitat connecting the crest of the Sierra with the Carson Range to the east. If pika response is governed by the temperature and dispersal thresholds observed in our study area, climate warming appears poised to cause extensive retraction and fragmentation of pika populations in the greater Lake Tahoe area within decades (bottom panels).

Availability of thermal refugia (minimum MST) within 4 km was the best predictor of site occupancy in our north Lake Tahoe study region (S3 Table). A simple logistic regression with this predictor strongly outperformed the next best model (ΔAICc = 2.4). Occupied and extirpated sites separate perfectly along this variable, such that all sites lacking refugia with MST cooler than 14.2°C within 4 km were extirpated, and all sites with cooler refugia supported extant pika populations (S3 Fig). From the historical (1910–1955) to current (2001–2010) period, warming temperatures resulted in 100% of sites in the Pluto triangle crossing from below to above this 14.2°C refuge threshold (S2 and S3 Figs). Using this model to project climatically suitable potential habitat area in the greater Lake Tahoe area, resulted in substantial contraction in climatically suitable area over time (Fig 4). Whereas current climate conditions result in 3,701 km^2^ of climatically suitable area within 4 km of refugial MST (< 14.2°C), the figure is projected to decline by 52% by 2030 (1,779 km^2^) and by 83% by 2050 (636 km^2^). The percent decline in the area of refugial conditions (MST < 14.2°C) from the current period baseline (1,214 km^2^) is projected to be 77% by 2030 (284 km^2^) and 97% by 2050 (33 km^2^).

## Discussion

We readily found remnant pika fecal pellets at all but one of 14 survey sites within the Pluto triangle, often in some abundance. Taluses in the Pluto triangle were 5–12 km away from the nearest known extant pika localities outside the triangle. This large volume and broad occurrence of evidence of former habitation, in combination with the considerable distances from occupied talus and intervening barriers, such as rivers, highways, and lower elevations, rule out the possibility that the pellets were deposited by isolated dispersers that lived out solitary lives in the Pluto triangle. We therefore conclude that the Pluto triangle was formerly inhabited by pikas. The 165-km^2^ area of the extirpated region is large relative to previously published accounts of modern and historical-era pika extirpations, which focused on 3-km-radius or smaller areas [24,27]. This extirpation echoes the prehistoric shifts of pika distribution, documented by Hafner [17,18] and Grayson [19], where substantial areas of landscape either were colonized by or became devoid of pikas when climate cooled or warmed, respectively.

From radiocarbon dates, we know that pikas were extirpated from the Pluto triangle sometime after 1958, with age ranges within the triangle spanning as late 1991. This is among the first accounts of a modern, climate-mediated extirpation of a species from within an area of contiguous distribution, resulting in fragmentation and loss of connectivity, as discussed below. While it is not possible to conduct an experiment on a singular event in the past, we believe the temporal association between this metapopulation extirpation and climate change, together with extensive literature empirically documenting pika vulnerability to climate change (e.g. physiology, behavior, paleontological, and historical distributional change) are compelling evidence of climate-mediated extirpation. An extensive search for empirical documentation of this type of phenomenon yielded only one study (fragmentation of sea ice habitat for polar bears) [13], though datasets used in other studies may also be suggestive of this type of event for other taxa [1,55]. We suspect that, while this phenomenon has not been frequently documented in the literature, it is likely already a commonplace phenomenon experienced by many species responding to climate warming [56].

Climate and weather-station data, in combination with our previously published model of pika site suitability, implicate climate change as the cause of the Pluto triangle extirpation. Weather station data show a substantial, 1.9°C, increase in temperature, with concomitant decline in snowpack (Fig 3, S1 Fig). The proportion of Pluto triangle sites with MST > 14.2°C, the mean MST at extirpated historical sites in California [27], has increased from 0% in the pre-1955 period to 100% of sites today. Higher MSTs appear to be linked to pika extirpations because they force pikas to reduce their foraging activity during daylight hours in order to avoid hyperthermia [16,57]. Subsurface talus temperatures may provide refuge from surface conditions, but unless vegetation is available to pikas below the talus surface [31] sub-surface refuge does not solve the problem of warm surface conditions reducing the animal’s ability to forage. The post-1955 period also saw the emergence of the first winters recorded with negligible snowpack (< 2 cm), including multiple consecutive year periods with negligible snowpack (e.g. 1959–1960, 1986–1990, 2013–2015; S1 Fig). Loss of snowpack has been hypothesized to be linked to extirpations because the loss of an insulating blanket of snow causes pikas to be exposed to extreme cold in winter [23,32,58]. Pikas, like other lagomorphs, do not hibernate and periods of extreme cold impose high metabolic costs and may lead to hypothermia [14,59]. Other potential drivers of the extirpation could include climate-mediated changes in forage quality [60] or land use change, concurrent with climate warming, and associated changes in community dynamics (e.g. predators, disease) [61,62], though we were unable to test these hypotheses.

The proportion of sites extirpated in this study exceeded the expected proportion based on climate and local habitat area, relative to other pika extirpations across California [27]. We propose that the accelerated extirpation could be due, in part, to declining climatic suitability of dispersal corridors connecting the triangle to adjacent habitat, and to the relatively low elevation of refugial mountain peaks within the Pluto triangle [22,24]. As temperature increased at high-elevation refugia, so did the temperature of low-elevation dispersal corridors, further isolating the Pluto triangle metapopulation. While nearby pika population centers are supported by more robust high-elevation refugia (e.g. Mount Rose, elevation 3,280 m, current MST = 11.2°C; Granite Chief Peak, 2,745 m, 13.2°C) that may act as source populations supporting lower elevation sites, the highest elevation refuge for pikas in the Pluto triangle is Mt Pluto (2,625 m, MST = 14.5°C). Our previously published model [27], premised on MST and talus area, did not effectively capture these nuances of metapopulation and habitat structure that may be relevant to the vulnerability of the Pluto triangle pika metapopulation to extirpation. In contrast, a simple model of thermal refuge of ~14°C MST threshold within a 4-km dispersal distance explains the extirpations of these sites well (S2 and S3 Figs). Similar, 4.5-km, dispersal thresholds for pikas have been found in recent genetic work [10,35]. The implication here is that when connectivity with higher elevation or cooler refugia is limited, local habitat abundance becomes less important in mediating persistence. In the absence of a rescue effect [63] pika persistence above an absolute temperature threshold may become precarious. Our previously published model may also underestimate temperature thresholds for pika persistence at sites with very high talus area, due in part to linear extrapolation of temperature thresholds to sites with high talus area [27].

A complementary hypothesis is that the regionally accelerated rate of extirpation could be partially attributed to local maladaptation favoring survival in cold, as opposed to warm, conditions. Moderate isolation, surrounding high-elevation source populations, and rapid climate warming from previously cold temperatures are all factors that could have contributed to local adaptation for survival in cold as opposed to the warm conditions now prevailing. Weather data from the Pluto triangle indicates that local temperatures increased at a rate of 1.9°C per century during the historical record, faster than the regional, Sierra Nevada-wide, rate of climate warming [47,51]. Species adapted for survival at high-elevations, like pikas, inhabit an environment of extreme weather fluctuations and face evolutionary trade-offs between being adapted to tolerate cold and hot conditions. The regional relative magnitude of these selective pressures may either predispose or disadvantage some populations for survival at warmer sites. A greater understanding of the species behavioral, physiological, and evolutionary adaptive capacity would help to elucidate its ability to cope with environmental change [64]. Adaptations to relatively cool conditions during much of the Holocene and Pleistocene periods may now be maladaptive.

Warming during the 20th century (Fig 3) was preceded by a long period of relative cold sometimes referred to as the Little Ice Age or the Matthes glacial advance(s), which lasted approximately from the 1400s to the latter 1800s or early 1900s [39,65,66], and corresponded to approximately a 0.2–2°C lowering of summer temperatures and a 90 m lowering in elevation of isotherms of mean temperature in the Sierra Nevada [39,67]. This long period of relative cold would have provided pikas with an extensive and contiguous region of pika-habitable temperatures in the Tahoe region (Fig 4).

Before their collapse, pika populations in the Pluto triangle region could be thought of as an isthmus of modest-elevation habitat connecting the “mainland” stronghold of pikas—the crest of the Sierra on the west side of Lake Tahoe—with a peninsula of pika habitat (Mount Rose/Carson Range) on the east side of Tahoe. The triangle was central to a contiguous area of pika distribution, with all but one habitat patch searched yielding evidence of previous occupancy. The regional pika population was contiguous in the sense that the maximum distance separating pika habitat patches across the triangle, from the Sierra crest to the Carson Range, was < 3 km, a distance across which pika dispersal has been empirically documented [68], and well below maximum estimated dispersal distances for the species [18,50]. It may have taken multiple generations of pika dispersal events to cross this corridor, but given widespread evidence of past occupancy, it is logical to conclude cooler conditions supported some degree of genetic connectivity between the Carson Range and the main crest of the Sierra Nevada. Rising "tides" of warm air have now “submerged” the Pluto triangle into more tenuous climate conditions for pikas and the isthmus has disappeared, resulting in habitat fragmentation and loss of metapopulation connectivity. Pikas in the Carson Range complex are now isolated from pikas on the main crest of the Sierra, by a distance of more than 25 km. Maximum dispersal distances for pikas over the last 6,000 years in the Rocky Mountains are estimated to be between 10 and 20 km [18,50]. Thus, the loss of this dispersal corridor suggests the complete loss of metapopulation and genetic connectivity through this corridor.

Our results suggest that, at least in some areas, the timeframe of shrinking of pika distribution as a result of warming temperatures is likely to be on the scale of decades, not centuries. If pika response to climate change in the greater Lake Tahoe area is governed by the dispersal and temperature thresholds observed in our study area, climate change could cause widespread mid-21^st^ century loss of pikas throughout much of the greater Lake Tahoe area (Fig 4). This study is among the first empirical examples of a modern, climate-mediated extirpation of a species from an area centrally located within contiguous habitat, resulting in habitat fragmentation. Habitat destruction and fragmentation has been widely documented as a cause of biodiversity loss [69] and both empirical and theoretical studies have demonstrated how fragmentation amplifies extinction risk from climate change [7,70]. Here we provide an example of the reverse: how climate change can also exacerbate habitat fragmentation. Our finding highlights how climate can interact with landscape and organismal factors to determine geographic patterns of range erosion under climate change. Collapsing sky-island systems, such as mountain-top metapopulations of pikas and other species (e.g., *Urocitellus beldingi* [71], *Tamias alpinus* [33]), should serve as living laboratories for understanding the mechanisms and dynamics by which metapopulation structure, habitat quality, immigration, emigration, genetic connectivity, and fragmentation interact with changing climate conditions to determine patterns of species distribution, abundance, and persistence. We anticipate this study foreshadows an era in which accelerating climate warming will drive the extirpations not just of species’ peripheral habitat, but cause fragmentation of core habitat for many species.

## Supporting information

S1 Fig Snow depth measurements for weather station at Tahoe City, CA, USA. Snow depth has decreased significantly over the period of record for snowpack, 1911–2015 (Spearman’s test, p < 0.0005, N = 91), with significantly lower snow depth in the years following 1955 than the preceding years (Mann-Whitney test, p < 0.0005). The frequency of years with negligible (< 2 cm) of snowpack increased from 0% of years before or during 1955 to 34% of years after (two-tailed z-test, p < 0.0005). Linear trend and confidence intervals are shown in blue and grey (linear regression, p < 0.01). Tahoe City forms the southwestern vertex of the Pluto triangle (Fig 1).
(TIF)

S2 Fig Graphical comparison of pika occupancy models. Formerly occupied Pluto triangle sites (N = 13) plotted against the California-wide model of pika occupancy (Stewart et al. [27]). Horizontal axis is refugial (minimum) MST within 1 km, following the California-wide model. Vertical axis is area of talus habitat within 1 km of the site centroid. Central dashed diagonal line represents 0.5 probability of occupancy as predicted by the California-wide model. Outer dashed lines represent 0.95 and 0.05 probability of occupancy. The proportion of sites extirpated within the Pluto triangle exceeded the expected proportions based on our previous model [27] (exact binomial test, p < 0.001). Dashed red line, MST = 14.2°C, is the mean temperature of extirpated historical sites in [27], and 0.5 probability of occupancy threshold for refugial (minimum) MST within 4 km for sites included in this paper (Fig 1, S1 and S3 Tables). A simple refugial MST model outperforms the California-wide model which incorporates talus habitat area within a 1-km radius, suggesting that when access to thermal refugia is limited, climate trumps habitat area as a driver of population persistence.
(TIF)

S3 Fig Pika occupancy status at north Lake Tahoe area pika sites as a function of the best performing predictor variable. North Lake Tahoe area pika sites are shown in Fig 1 and listed in S1 Table (N = 38). Extant and extirpated sites differentiate perfectly by this metric (Welch’s t-test, p < 0.005). Four kilometers represents a threshold above which pika dispersal becomes increasingly limited (Tapper, 1973; Hafner, 1994; Hafner & Sullivan, 1995; Castillo et al. 2016). Fourteen degrees Celsius MST represents a threshold above which pika persistence becomes more tenuous (Stewart et al., 2015). Dashed grey line (MST = 14.2°C) is the 0.5 probability of occupancy as modeled by logistic regression at these sites (S3 Table). Boxes are interquartile range. Historical period is 1910–1955, current period is 2001–2010.
(TIF)

S1 Table Locations and data for north Lake Tahoe area talus surveys. Pika sign abbreviations: A–audio detection, GH–green haypile, FP–fresh pellets, BP–buried pellets (found in accumulations of organic debris), SP–surface pellets, not fresh (found on rock surfaces below other rocks), V–visual detection. Occurrence coordinates for survey data provided by the Nevada Department of Wildlife are obscured to the level of a Public Land Survey System section at their request. Pika presence at Mount Rose 1 was reported by M. M. Peacock (University of Nevada, Reno); Tamarack Pk 1 by C. I. Millar (US Forest Service, Pacific Southwest Research Station); and Rose Knob Pk 3 by C.R. Parker. See also Fig 1.
(XLSX)

S2 Table Radiocarbon data for buried pika scat. Uncertainty is expressed as standard deviation. Calibrated age ranges represent 95% confidence intervals.
(XLSX)

S3 Table AICc model comparison for north Lake Tahoe area sites. AICc model comparison of 10 models of pika site occupancy in the north Lake Tahoe area. MST_min_1k is minimum mean summer temperature within 1 km of the site centroid; log_talus_area is log transformed talus area within 1 km of the site centroid.
(XLSX)
