# Supplementary figures and images for: Apparent climate-mediated loss and fragmentation of core habitat of the American pika in the Northern Sierra Nevada, California, USA
Source: PLoS One. 2017 Aug 30;12(8):e0181834. doi: 10.1371/journal.pone.0181834 (PMC5576638; doi:10.1371/journal.pone.0181834)

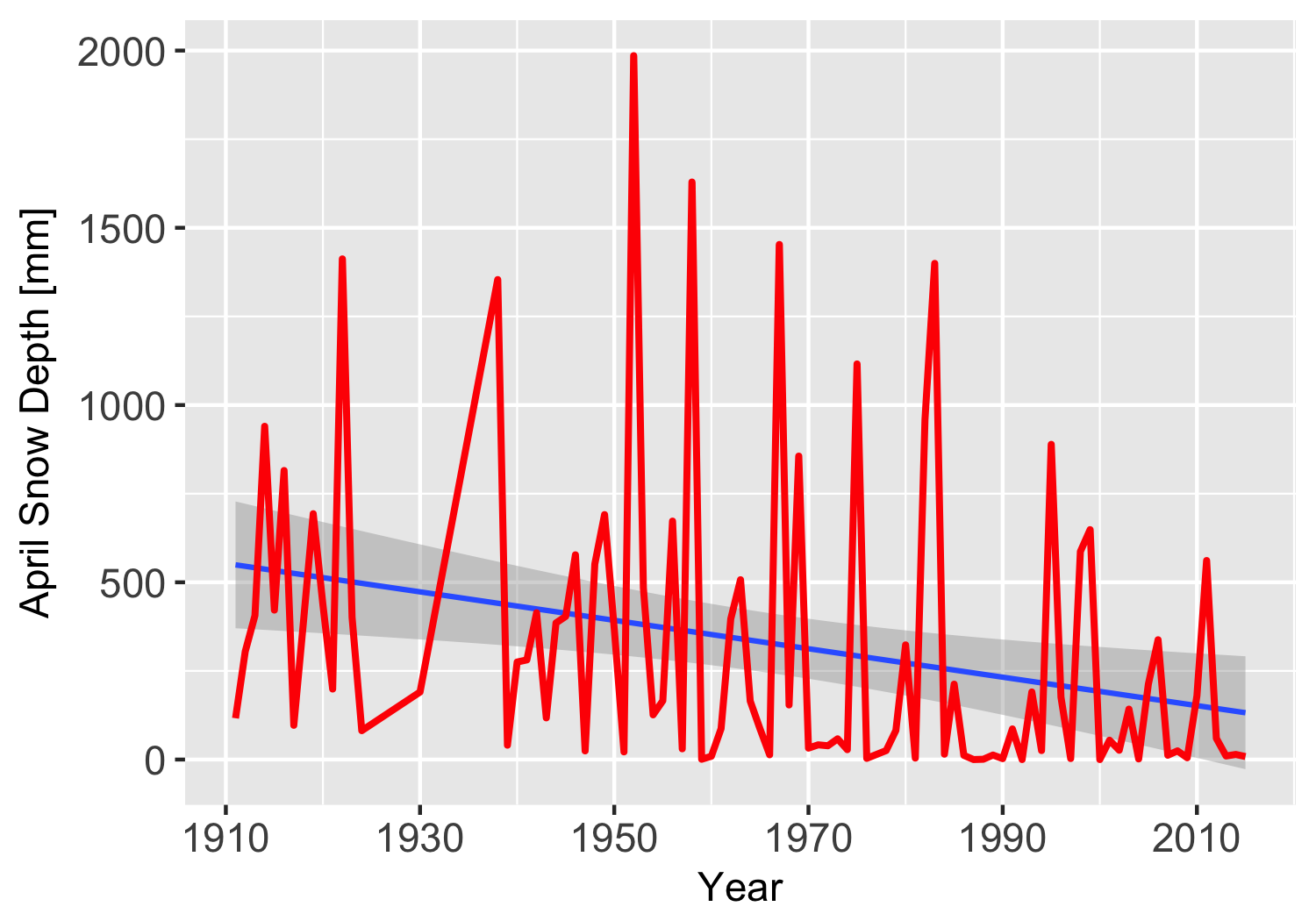

Supplement: S1 Fig — Snow depth has decreased significantly over the period of record for snowpack, 1911–2015 (Spearman’s test, p < 0.0005, N = 91), with significantly lower snow depth in the years following 1955 than the preceding years (Mann-Whitney test, p < 0.0005). The frequency of years with negligible (< 2 cm) of snowpack increased from 0% of years before or during 1955 to 34% of years after (two-tailed z-test, p < 0.0005). Linear trend and confidence intervals are shown in blue and grey (linear regression, p < 0.01). Tahoe City forms the southwestern vertex of the Pluto triangle (Fig 1). (TIF) [file pone.0181834.s001.tif]

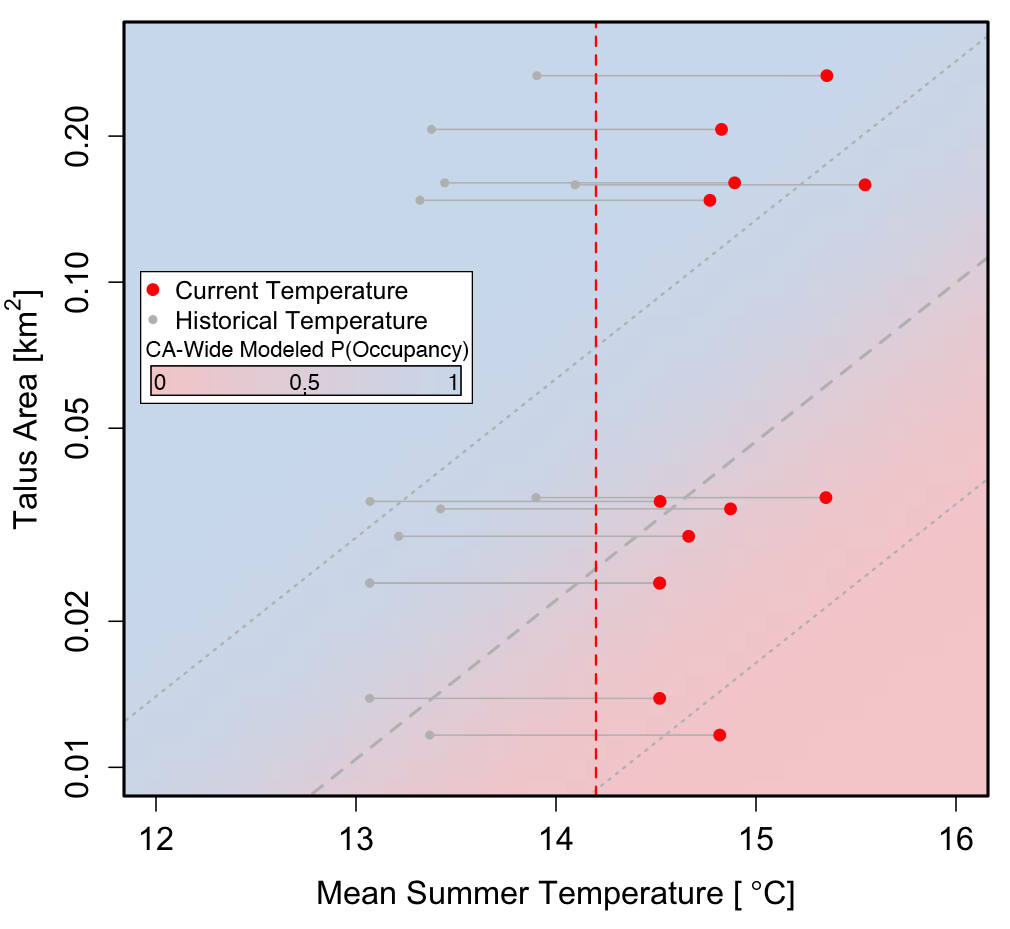

Supplement: S2 Fig — Formerly occupied Pluto triangle sites (N = 13) plotted against the California-wide model of pika occupancy (Stewart et al. [27]). Horizontal axis is refugial (minimum) MST within 1 km, following the California-wide model. Vertical axis is area of talus habitat within 1 km of the site centroid. Central dashed diagonal line represents 0.5 probability of occupancy as predicted by the California-wide model. Outer dashed lines represent 0.95 and 0.05 probability of occupancy. The proportion of sites extirpated within the Pluto triangle exceeded the expected proportions based on our previous model [27] (exact binomial test, p < 0.001). Dashed red line, MST = 14.2°C, is the mean temperature of extirpated historical sites in [27], and 0.5 probability of occupancy threshold for refugial (minimum) MST within 4 km for sites included in this paper (Fig 1, S1 and S3 Tables). A simple refugial MST model outperforms the California-wide model which incorporates talus habitat area within a 1-km radius, suggesting that when access to thermal refugia is limited, climate trumps habitat area as a driver of population persistence. (TIF) [file pone.0181834.s002.tif]

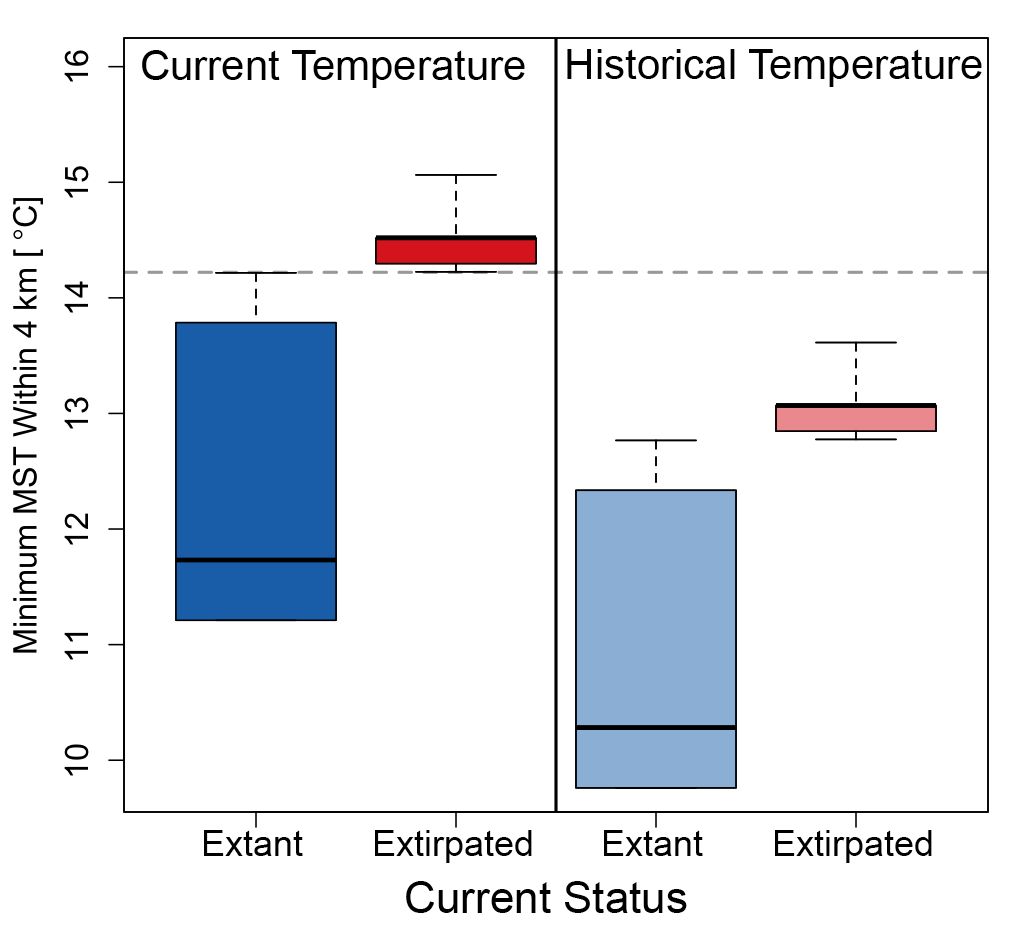

Supplement: S3 Fig — North Lake Tahoe area pika sites are shown in Fig 1 and listed in S1 Table (N = 38). Extant and extirpated sites differentiate perfectly by this metric (Welch’s t-test, p < 0.005). Four kilometers represents a threshold above which pika dispersal becomes increasingly limited (Tapper, 1973; Hafner, 1994; Hafner & Sullivan, 1995; Castillo et al. 2016). Fourteen degrees Celsius MST represents a threshold above which pika persistence becomes more tenuous (Stewart et al., 2015). Dashed grey line (MST = 14.2°C) is the 0.5 probability of occupancy as modeled by logistic regression at these sites (S3 Table). Boxes are interquartile range. Historical period is 1910–1955, current period is 2001–2010. (TIF) [file pone.0181834.s003.tif]
